# Great fraction of dissolved organic C and N in the primary per-humid *Chamaecyparis* forest soil

**DOI:** 10.1186/s40529-015-0106-6

**Published:** 2015-09-30

**Authors:** Chih-Wei Tsai, Guanglong Tian, Chih-Yu Chiu

**Affiliations:** 1 Biodiversity Research Center, Academia Sinica, Taipei, 11529 Taiwan; 2 Environmental Monitoring and Research Division, Monitoring and Research Department, Metropolitan Water Reclamation District of Greater Chicago (MWRD), Lue-Hing R&D Laboratory, 6001 W. Pershing Road, Cicero, IL 60804 USA

**Keywords:** Labile resource, DOC, DON, Ammonium, Nitrate

## Abstract

**Background:**

Labile organic matter plays a crucial role in a variety of forest functions, however, our understanding to its quality and quantity across various forests is limited, particularly primary forests. We investigated soil labile C and N (i.e. microbial biomass C and N, dissolved organic carbon (DOC) and nitrogen (DON), associated ammonium, and nitrate) at three topographic locations (i.e. summit, footslope and lakeshore) in a primary *Chamaecyparis* forest of Taiwan. The following hypotheses are tested in this study: (1) This undisturbed *Chamaecyparis* forest shows the great size of soil labile C and N; (2) there is an evident topographic effect on the distribution of soil labile C and N and the associated inorganic N over seasons.

**Results:**

Fulfilling with our first hypothesis, the considerable size of labile C and N in this forest soil was quantified. Abundant C availability and the acidity of soils in this forest favoured ammonium production over nitrate. The undisturbed environment with per-humid and acidic soil was linked to the high concentrations of soil DOC and DON as the dominant form in N dynamics. In contrast to our second hypothesis, topographic effects on soil labile C and N were generally not evident, suggesting the homogeneous soil environment across various topographic locations in this *Chamaecyparis* forest.

**Conclusions:**

This study illustrates the sustainable importance of primary montane forests for being sources of DOC and DON.

## Background

Carbon and nitrogen play essential roles in developing, maintaining and reproducing of organisms. Transformation, movement and reuse of these nutrients are important to sustain a variety of ecosystem functions (Galloway et al. 2004). However, our understanding to the quality and quantity of soil labile C and N resources across various forests is still limited, in particular, subtropical primary montane forests. The ecological importance of soil organic matter lies in its biodegradability and availability for microbes, fauna and plant (Ghani et al. 2007). Thus, the comprehension of the size of soil labile C and N resources can help us understand the fraction of C and N immediately available to soil organisms and cycling in amount of C and N resources in forest ecosystems. Of these, microbial biomass C (C_mic_) and N (N_mic_) are the most labile C and N pools in soils. Although they generally constitute only 1–3 % and 1–5 % of total soil C and N (Moore et al. 2000), they are related to the size of decomposed organic material and soil physical and chemical properties.

Neff and Asner (2001) noted dissolved organic carbon (DOC) and dissolved organic nitrogen (DON) constitute the main fraction of available monomers for microorganisms. The quantity of DOC can impact the heterotrophic microbial activity and the associated N transformation (Singh and Kashyap 2006). The proportion of DON and inorganic N can be an important indicator of water quality and nutrients in hydrological cycling (Fang et al. 2009; Lutz et al. 2011). With the shift of nitrogen cycling in forest ecosystems from organic to inorganic form, recent studies have shown that N can be lost from temperate forest ecosystems predominantly by the way of DON (Hedin et al. 1995; Perakis and Hedin 2002). So far, studies addressing the dynamics of labile C and N have mainly focused on temperate forests, but the potential contribution of subtropical primary forest soils to C and N loadings is still poorly known.

*Chamaecyparis* forests are valuable natural resources in eastern Asia because of its high quality timber, control of soil erosion, and storage of C and N. Much effort has been invested in investigating soil properties and fertility management of Japanese hinoki cypress (*Chamaecyparis obtusa*) plantations (e.g. Inagaki et al. 2008, 2011). By contrast, characteristics of soil organic matter under natural *Chamaecyparis* forests in subtropical montane area are not well known. Today, the *Chamaecyparis* forest in the Chi-Lan Mountain is one of the few preserved primary *Chamaecyparis* forests in northcentral Taiwan. The unique aspect of the *Chamaecyparis* forest is that it has the environment, which is rarely disturbed, high precipitation, and mild temperature.

Previous studies have shown that strongly acidic and wet environment resulted in the low diversity of soil bacterial communities in this region (Lin et al. 2010). The litter decomposition and humification in the *Chamaecyparis* forest is generally very slow (Chen et al. 2013; Chung et al. 2012), and this may result in the considerable soil organic matter and the great size of labile C and N loadings. Chen and Chiu (2000) investigated soil structure across a slope in the *Chamaecyparis* forest and found out a common phenomenon—a thick layer of organic horizon (i.e. 10–30 cm) derived from *Sphagnum* on the surface of soils. However, little is known about the size of the soil labile C and N in this primary *Chamaecyparis* forest. Additionally, Schmidt et al. (2010) demonstrated that DOC and DON were the predominant forms of C and N resources released from surface soils in a secondary *Chamaecyparis* forest of Taiwan. The objectives of the study were therefore to quantify concentrations of soil labile C and N under the *Chamaecyparis* forest and evaluate their distributions along topographic locations over seasons.

## Methods

### Study area

**Fig. 1:**
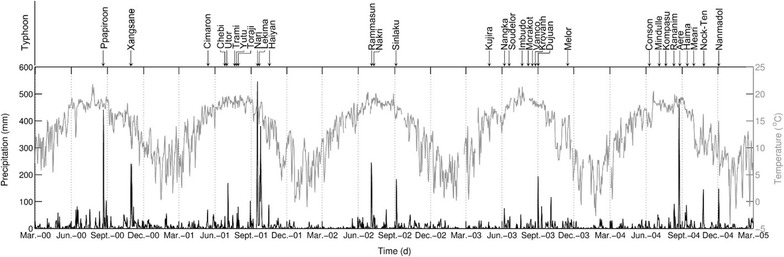
Daily temperature and daily precipitation

Surrounding a small and shallow lake, the primary *Chamaecyparis* forest is located in the Yuan Yang Lake Conserve area (24°35′N, 121°24′E). The Lake is small (3.6 × 10^4^ m^2^) and shallow (4.5 maximum depth). The annual precipitation is over 4000 mm and the annual mean temperature is 12 °C in this region. Daily precipitation illustrates clear pulse during summer, mainly driven by typhoons (Fig. 1). *Chamaecyparis obtusa* var. *formosana* (Hayata) Rehder is the dominant species of this Taiwan cypress forest. As a result of high annual precipitation soils are permanently moist and acidic (i.e. pH <4) (Lin et al. 2011). Although this area is geographically belonged to subtropical zone, this primary montane forest, to some extent, can be analogous to temperate rainforests due to its climatic characteristics.

### Sampling locations

**Fig. 2:**
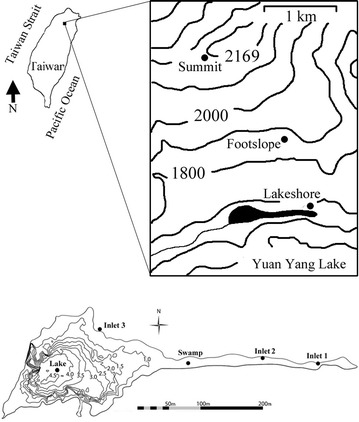
Map of sampling sites at Yuan Yang Lake Nature Reserve, at the boundary point of Taoyuan and Ilan Counties, Taiwan

A slope about 28° next to the lake was divided into three sampling locations, summit, footslope and lakeshore (Fig. 2). The soil at summit is dominantly Albaquult and relatively well drained, whereas the soil at lakeshore is dominantly Histosol and poorly drained due to the frequent immersion by lake water. Footslope, between summit and lakeshore, is dominantly Dystrochrept. The thick layer of organic horizon was derived from *Sphagnum* across three locations. The depth of organic horizon decreased with an increase in the elevation of the slope for which the depth of organic horizon for lakeshore, footslope and summit was about 30, 15 and 10 cm respectively. The depth of A horizon was small and ranged between 1 and 5 cm across three locations (Chen and Chiu 2000).

### Soil sampling and processing

Five replicates of sampling plots (50 × 50 m each) at each sampling location were evenly chosen along a transect vertical to the slope. After carefully removing undecomposed litter, soil samples at 0–10 cm deep layer (O/A horizon) were collected using a soil auger (8 cm in diameter). The O/A horizon refers to the surface soil which was dominated with organic matter and humus. For lake water samples, each 100 ml water sample was collected from three inlet, one swamp (i.e. shallow) and one lake (i.e. deep) locations (Fig. 2). Three replicates were collected at each location. All samples were brought back to laboratory and kept at 4 °C before analysis. The soil subsamples were air-dried and ground to pass a 2.0 mm sieve for TC and TN analysis.

### Laboratory analysis

Soil moisture was determined by oven-dried at 105 °C. The C_mic_ were determined by fumigation extraction method with a conversion factor of 2.22 (Wu et al. 1990). The N_mic_ were estimated from ninhydrin-reactive N released from the biomass and then determined using colorimetrically at 560 nm (Amato and Ladd 1988). Five sets of soil subsamples from each sampling location were incubated at 25 °C and 55 % soil moisture for 7 days for determination of net N mineralisation and net N nitrification rates (Hart and Binkley 1985). For DOC, DON and inorganic N, water extractions were made from all samples. Inorganic nitrogen was determined by using 25 g soil samples with 0.5 M K_2_SO_4_ extraction, followed by colorimetry flow injection analysis for ammonia and nitrate (FIA; Quikchem-Method-10-107-04-1-L, 1999 and Quikchem-Method-10-107-06-2-A, 1997, respectively). An in-line persulfate digestion followed by FIA in the same auto-analyser was applied in order to determine the concentration of the total dissolved nitrogen, TDN (Quikchem-Method-10-107-04-3-P, 2000). The concentration of the dissolved organic nitrogen (DON) was estimated as difference between TDN and inorganic N. The amount of extractable DOC was evaluated using an Aurora 1030 Wet Oxidation TOC Analyser (O.I. Analytical, USA). TC and TN in soil were analysed by high temperature combustion on a Fisons NA-1500 NCS Analyser (Italy). Water sample was analysed for DON, NH_4_^+^ and NO_3_^−^ using the same procedure as soil samples but without extraction processes. All samples were analysed within 2 weeks of sample collection.

### Data analysis

Linear mixed-effects models were used to assess the effect of ‘Location’ (i.e. summit, footslope, lakeshore) on the concentration of TC, TN, C_mic_, N_mic_, DOC, DON, ammonium, and nitrate for each sampling season. Additionally, linear mixed-effects models were also used to assess the effect of ‘Location’, ‘Season’, and an interactive effect of ‘Location’ and ‘Season’ on soil moisture and soil nutrients across multiple sampling years. Replicate sampling plot (Replication), and replicate sampling plot nested within each sampling year (Year/Replication) were set up as the random effect for former and latter models, respectively. Models used *p* value calculated based on Satterthwaite’s approximations to indicate the significance of the interested fixed effects. Statistical analyses were carried out using R (R Core Team 2013).

## Results

### Soil total C and N and microbial biomass

**Fig. 3:**
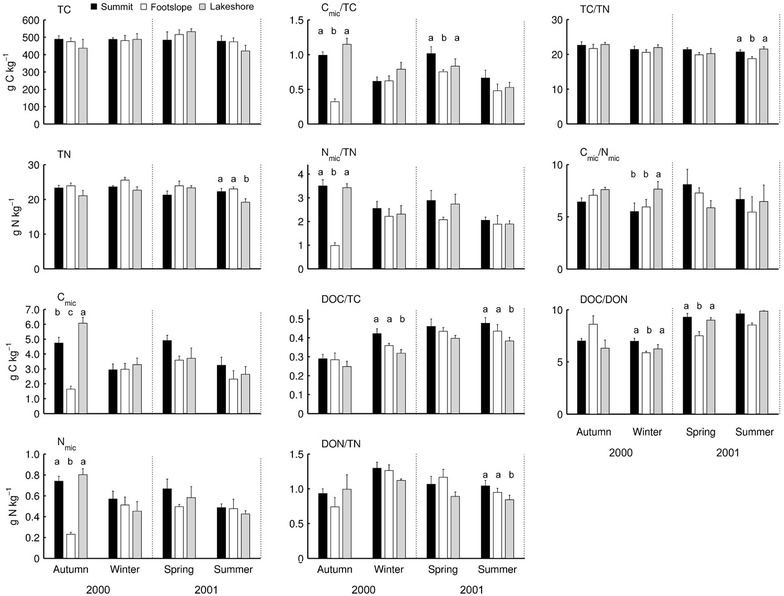
The concentration of total C (TC), total N (TN), microbial biomass C (C_mic_) and microbial biomass N (N_mic_), dissolved organic C (DOC), dissolved organic N (DON), and associated ratios at three topographic locations in 2000 autumn and winter and in 2001 spring and summer. *Different letters* indicate significant difference among locations for a given season (*p* < 0.05)

Seasonal concentrations of soil TC, TN, C_mic_, N_mic_ and the ratio of C_mic_/TC, N_mic_ /TN, DOC/TC, DON/TN, TC/TN, C_mic_/N_mic_, and DOC/DON are presented in Fig. 3. The size of soil TC and TN was high, ranging between 421 and 532 g C kg^−1^ and between 19 and 26 g N kg^−1^, respectively. Similarly, the size of soil C_mic_ and N_mic_ was also very high, ranging between 1.6 and 4.9 g C kg^−1^ and between 0.2 and 0.7 g N kg^−1^, respectively. Resulting from very high amount of soil TC and TN, the ratio of soil C_mic_/TC and N_mic_/TN was low, between 0.3 and 1.1 and between 1.0 and 3.5. The ratio of soil DOC/TC and DON/TN was also low, between 0.2 and 0.5 and between 0.1 and 1.3. The ratio of soil TC/TN, C_mic_/N_mic_, and DOC/DON across three topographic locations was between 18.7 and 22.8, between 5.4 and 8.1, and between 5.9 and 9.9, respectively.

### Soil moisture and the concentrations of soil DOC, DON, ammonium, and nitrate

**Fig. 4:**
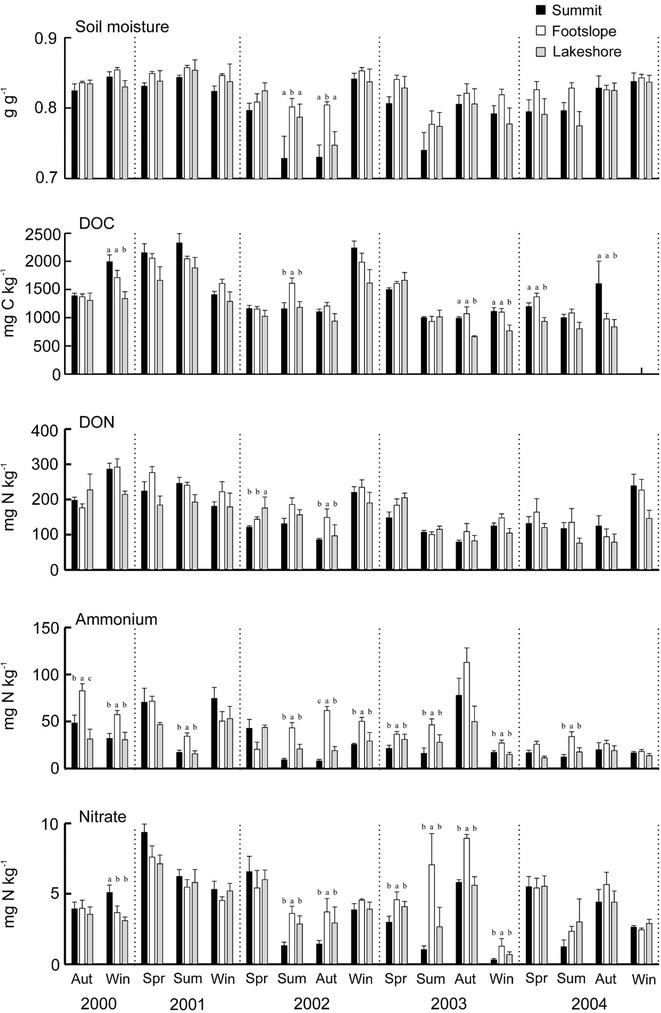
Soil moisture and concentration of dissolved organic C (DOC), dissolved organic N (DON), ammonium, and nitrate of O/A horizon at three topographic locations. *Different letters* indicate significant difference among locations for a given season (*p* < 0.05)

Soil moisture levels were always high throughout the year at all topographic locations (Fig. 4). Seasonal concentrations of soil DOC, DON, ammonium and nitrate are presented in Fig. 4. The concentrations of soil DOC and DON were high, ranging from 900 to 1946 mg C kg^−1^ and 90 to 215 mg N kg^−1^. For soil inorganic nitrogen, the concentration of soil ammonium, ranging from 16 to 80 mg N kg^−1^, was higher than nitrate. Soil nitrate, occupying the smallest part of soil inorganic N, ranged from 0.8 to 6.8 mg N kg^−1^.

### Soil nitrogen mineralisation and nitrification

**Fig. 5:**
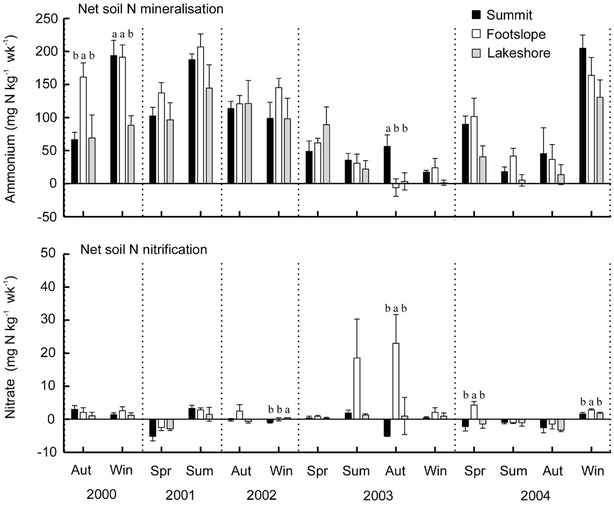
Soil net N mineralisation and nitrification at three topographic locations*. Different letters* indicate significant difference among locations for a given season (*p* < 0.05)

The N mineralisation in this forest soil was evident. During a week incubation, the ammonium produced by N mineralisation reached 131 mg N kg^−1^. In contrast, the nitrification (measured as nitrate production during the incubation) was negligible (Fig. 5).

### The concentration of DON, ammonium, and nitrate of lake water

**Fig. 6:**
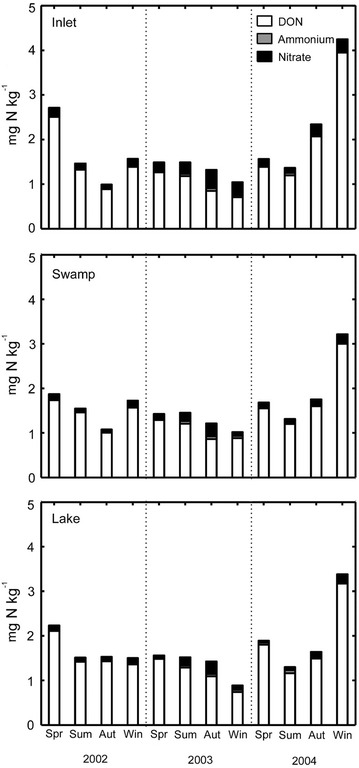
Average concentrations of DON, NH_4_^+^ and NO_3_^−^ of the lake water

The concentration of DON of lake water ranged between 0.77 and 3.37 mg N kg^−1^, and the concentrations of ammonium and nitrate were relatively low compared to the DON (Fig. 6). Thus, organic N was the dominant form of N in lake water for which DON contributed to the high proportion (70–93 %) of the total N in lake water.

### Topographic, seasonal, and interactive effects on soil nutrients

General speaking, the topographic effect on DOC and DON was not evident, but there were some significant cases over seasons. Surface soils at footslope had slightly higher moisture over seasons (Table 1). The soil DON concentration at footslope was highest over seasons, and that at lakeshore was lowest in winter (Table 1). Surface soils at footslope had the highest ammonium and nitrate concentrations in summer and autumn. Looking at the seasonal pattern, soil moisture was lowest in summer (Table 1). The soil DON concentration was highest in winter and lowest in autumn (Table 1). The ammonium concentration was lowest in summer and the nitrate concentration was lowest in spring, and the ammonium production through net N mineralisation processes was greatest in winter (Table 1).

**Table 1:** Linear mixed-effects model analysis: the effects of topographic location and seasons on soil moisture, DOC, DON, NH_4_^+^, NO_3_^−^ concentrations, net N mineralisation, and net N nitrification in O/A horizon

Effects	Soil moisture (g g^−1^)		DOC (mg C kg^−1^)		DON (mg N kg^−1^)		NH_4_^+^ (mg N kg^−1^)		NO_3_^−^ (mg N kg^−1^)		Net N mineralisation (mg N kg^−1^ wk^−1^)		Net N nitrification (mg N kg^−1^ wk^−1^)	
	Coeff.	SE	Coeff.	SE	Coeff.	SE	Coeff.	SE	Coeff.	SE	Coeff.	SE	Coeff.	SE
Location														
Summit^a^	0	0	0	0	0	0	0	0	0	0	0	0	0	0
Footslope	0.02*	0.01	NS	–	35.70*	16.78	NS	–	NS	–	NS	–	NS	–
Lakeshore	NS	–	NS	–	NS	–	NS	–	NS	–	NS	–	NS	–
Season														
Spring^a^	0	0	0	0	0	0	0	0	0	0	0	0	0	0
Summer	−0.03**	0.01	NS	–	NS	–	−24.20***	6.69	−3.62***	0.64	NS	–	NS	–
Autumn	NS	–	NS	–	−42.58*	17.11	NS	–	−1.65*	0.65	NS	–	NS	–
Winter	NS	–	NS	–	38.04*	16.07	NS	–	−2.60***	0.61	45.83*	20.45	NS	–
Interaction														
Footslope × Summer	NS	–	NS	–	NS	–	24.63**	9.46	2.50**	0.91	NS	–	NS	–
Lakeshore × Summer	NS	–	NS	–	NS	–	NS	–	NS	–	NS	–	NS	–
Footslope × Autumn	NS	–	NS	–	NS	–	31.67***	9.34	2.02*	0.90	NS	–	NS	–
Lakeshore × Autumn	NS	–	NS	–	NS	–	NS	–	NS	–	NS	–	NS	–
Footslope × Winter	NS	–	NS	–	NS	–	NS	–	NS	–	NS	–	NS	–
Lakeshore × Winter	NS	–	NS	–	−60.05**	22.86	NS	–	NS	–	NS	–	NS	–

Coeff. and SE are the estimated coefficient and standard error of each independent variable in the model

The + or − before the value of Coeff. indicates the higher or lower value than ‘Summit’ in location or ‘Spring’ in season

*** *p* < 0.001, ** *p* < 0.01, * *p* < 0.05, NS not significant (*p* >  0.1)

^a^Set to zero due to being the standard for comparison within each categorical factor

## Discussion

### The concentration of TC, TN and labile C and N resources

The high liable C and N concentrations in soils might be related to the characteristics of this primary *Chamaecyparis* forest. This primary *Chamaecyparis* forest has accumulated abundant dead woody debris on forest floor (Jien et al. 2010), suggesting a great size of C and N pools in this forest (Gonzalez-Polo et al. 2013; Hafner et al. 2005; Wirth et al. 2009). This may partly explain high soil TC and TN concentrations in this *Chamaecyparis* forest. Long-standing fog immersion has created the suitable environment for epiphytic bryophytes to thrive (Chang et al. 2002), and the cryptogamic cover, such as algae, fungi, lichens and bryophytes, is essential for the C and N cycle in forest ecosystems (Elbert et al. 2012), suggesting that the high amount of organic C and N fluxes might be also associated with the appearance of abundant bryophytes in this primary *Chamaecyparis* forest. A relatively low ratio of C_mic_/TC and N_mic_/TN suggests that C and N resources used by microbes were limited in the forest, and this could be due to the low diversity of soil bacterial communities under the acidic soil environments (Lin et al. 2010), and the constraint of the year-around per-humid environment might also hinder microbial activity and productivity.

The DOC and DON concentrations were profoundly high in the *Chamaecyparis* forest compared to those in the temperate deciduous forest (DOC: 150–225 mg C kg^−1^; DON: 35 mg C kg^−1^) in Hokkaido, Japan (Shibata et al. 2013) and the temperate coniferous (DON: 7–10 mg C kg^−1^) and deciduous forest (DON: 6–7 mg-C kg^−1^) in Thuringia forest, Germany (Zhong and Makeschin 2003). The fluxes of DOC and DON in forest floor leachates have been shown to be positively related to the amount of annual precipitation (Michalzik et al. 2001), suggesting that the high fluxes of dissolved organic matter in this *Chamaecyparis* forest soils might be associated with the high annual precipitation. Additionally, canopy and forest floor have been identified as the important sources of DON, in particular, in pristine ecosystems (Solinger et al. 2001). Schmidt et al. (2010) found that the DOC fluxes (478–962 kg C ha^−1^ yr^−1^) in forest floor leachates were profoundly high and DON (8–16 kg N ha^−1^ yr^−1^) was the major form of total N released from a secondary *Chamaecyparis* forest near our primary forest. We also observed that the O/A horizon of the *Chamaecyparis* forest soils contained the great amount of DOC (900–1946 mg C kg^−1^), and DON was the dominant forms of N resources, suggesting the existence of high DOC and DON fluxes in this primary forest soil. In addition, we also found that DON being the predominant form of N and in lake water for which DON contributed very high proportions (70–93 %) of total dissolved N. Similarly, this phenomenon was also found in some temperate undisturbed rainforests (Hedin et al. 1995; Perakis and Hedin 2002).

### Soil nitrogen mineralisation and nitrification

Compared to DON, ammonium and nitrate concentrations were relatively low at our study forest, but the magnitude was similar to the amount of other forests (Ge et al. 2014; Montaño et al. 2007; Shibata et al. 2013). With negligible amounts of external inputs of ammonium and nitrate (Chang et al. 2002), the most inorganic nitrogen in the forest was the product transformed from organic N. Montaño et al. (2007) demonstrated that the great size of DOC can stimulate heterotrophic microbes and N mineralisation, suggesting that the high ammonium production in this *Chamaecyparis* forest might be associated with its high C resources. Furthermore, nitrate may be easily lost from soils through direct absorption by plants and leaching processes due to its high mobility, and hence wet environments in such a high rainfall area may also contribute to the low nitrate concentration in surface soils. Immobilisation is one of the important processes in N dynamics. However, the ratio of C/N (i.e. TC/TN) of the O/A horizon was just 20, which was not higher than the critical level of 25 for N immobilisation, the N immobilisation should not be so intense in this forest. Acidity, in particular pH <5.5, has a detrimental effect on the nitrifying bacteria, thus reducing nitrification efficiency (Ste-Marie and Paré 1999). The soil pH was below 4, and the variation of pH was small (i.e. 3.3–3.5) in this *Chamaecyparis* forest soil. This might have an effect on low nitrifying activity and result in low amount of nitrate production.

### Topographic effect and seasonal variation

Our second hypothesis was not well supported—as the topographic effects on labile C and N and the inorganic N was not consistently evident over seasons. No apparent differences in microbial biomass among soils at various locations suggest that the soil environment of the entire forest across topography was similar. Only few cases in specific seasons could be detected. For example, DON concentrations were relatively low at lakeshore in particular in winter, and this was probably associated with the flooding events which brought organic matter away (Chung et al. 2012). We also observed that soils at footslope had highest inorganic N concentrations in rainy seasons (i.e. summer and autumn), and this might be partly due to the draining environment at footslope where soils can hold more water to increase mineralisation efficiency. The great amount of precipitation and relative warm environments in summer suggests that soils might experience intense dry-wet processes, and this probably stimulated soil microbes to greatly utilise organic N resources in soils. According to previous studies in this area (Chen and Chiu 2000), the detectable migration of humus and top soils from summit to footslope only had marginal effects on the distribution of labile nutrients, suggesting that the topographic effect of humus migration on soil nutrients might not be significant.

## Conclusions

This subtropical primary montane forest has accumulated the considerable size of DOC in the O/A horizon. DON was the predominant form of total dissolved N resources in soils and lake water. Topographic effects on soil nutrients were not very significant, suggesting that the dynamics of soil nutrient components was relatively equitable across topography, and this may be due to the strong control of per-humid climate and acidic soil conditions. These findings reveal the subtropical primary montane forest soils may hold the great amount of DOC and DON resources, suggesting the importance of this primary montane forest in regional C and N biogeochemical cycles.
